# Proteomic Analysis of *Biomphalaria glabrata* Hemocytes During *in vitro* Encapsulation of *Schistosoma mansoni* Sporocysts

**DOI:** 10.3389/fimmu.2018.02773

**Published:** 2018-11-29

**Authors:** Nathalie Dinguirard, Marília G. S. Cavalcanti, Xiao-Jun Wu, Utibe Bickham-Wright, Grzegorz Sabat, Timothy P. Yoshino

**Affiliations:** ^1^Department of Pathobiological Sciences, University of Wisconsin, Madison, WI, United States; ^2^Department of Physiology and Pathology, Federal University of Paraíba, João Pessoa, Brazil; ^3^Biotechnology Center/Proteomics-Mass Spectrometry Facility, University of Wisconsin, Madison, WI, United States

**Keywords:** innate immunity, mollusk, *Biomphalaria glabrata*, *Schistosoma mansoni*, hemocytic encapsulation, sporocyst, proteomic response, *in vitro* cytotoxicity assay

## Abstract

Circulating hemocytes of the snail *Biomphalaria glabrata*, a major intermediate host for the blood fluke *Schistosoma mansoni*, represent the primary immune effector cells comprising the host's internal defense system. Within hours of miracidial entry into resistant *B. glabrata* strains, hemocytes infiltrate around developing sporocysts forming multi-layered cellular capsules that results in larval death, typically within 24–48 h post-infection. Using an *in vitro* model of hemocyte-sporocyst encapsulation that recapitulates *in vivo* events, we conducted a comparative proteomic analysis on the responses of hemocytes from inbred *B. glabrata* strains during the encapsulation of *S. mansoni* primary sporocysts. This was accomplished by a combination of Laser-capture microdissection (LCM) to isolate sections of hemocyte capsules both in the presence and absence of sporocysts, in conjunction with mass spectrometric analyses to establish protein expression profiles. Comparison of susceptible NMRI snail hemocytes in the presence and absence of sporocysts revealed a dramatic downregulation of proteins in during larval encapsulation, especially those involved in protein/CHO metabolism, immune-related, redox and signaling pathways. One of 4 upregulated proteins was arginase, competitor of nitric oxide synthetase and inhibitor of larval-killing NO production. By contrast, when compared to control capsules, sporocyst-encapsulating hemocytes of resistant BS-90 *B. glabrata* exhibited a more balanced profile with enhanced expression of shared proteins involved in protein synthesis/processing, immunity, and redox, and unique expression of anti-microbial/anti-parasite proteins. A final comparison of NMRI and BS-90 host hemocyte responses to co-cultured sporocysts demonstrated a decrease or downregulation of 77% of shared proteins by NMRI cells during encapsulation compared to those of the BS-90 strain, including lipopolysaccharide-binding protein, thioredoxin reductase 1 and hemoglobins 1 and 2. Overall, using this *in vitro* model, results of our proteomic analyses demonstrate striking differences in proteins expressed by susceptible NMRI and resistant BS-90 snail hemocytes to *S. mansoni* sporocysts during active encapsulation, with NMRI hemocytes exhibiting extensive downregulation of protein expression and a lower level of constitutively expressed immune-relevant proteins (e.g., FREP2) compared to BS-90. Our data suggest that snail strain differences in hemocyte protein expression during the encapsulation process account for observed differences in their cytotoxic capacity to interact with and kill sporocysts.

## Introduction

Circulating hemocytes of *Biomphalaria* snail species represent the primary immune effector cells of the host, and, when encountering primary sporocysts of incompatible strains of the human blood fluke *Schistosoma mansoni*, respond by rapid encapsulation and destruction of early developing larvae (1–5). Cellular encapsulation responses typically are characterized by infiltration of circulating hemocytes around the newly transforming primary sporocysts, commencing usually within 2–3 h post-infection, followed by adherence and spreading of hemocytes in multiple layers around the parasite that result in the killing of encapsulated larvae within 24–48 h post-infection (6, 7). An *in vitro* model of larval encapsulation has been developed for the *B. glabrata-S. mansoni* system that closely parallels the events observed under natural *in vivo* infection conditions (8). In this cell-mediated cytotoxicity (CMC) assay, live *in vitro* transformed primary sporocysts (9) were co-incubated in 1-mL tubes with freshly extracted hemolymph isolated from inbred susceptible and resistant strains of *B. glabrata*, resulting in the formation hemocytic capsules around sporocysts. Although hemocytes of both susceptible and resistant snail strains formed cellular encapsulations, significantly enhanced killing/damage of sporocysts was observed in reactions with resistant snail cells when compared to those involving hemocytes from susceptible snails. Follow-up experiments showed that components from plasma (cell-free hemolymph) alone also may be involved in triggering the CMC response, although the humoral factor(s) and their functional role in hemocyte reactivity have yet to be identified and described (7, 10). Additional support that hemocytes are the primary mediators of anti-schistosome resistance recently has been demonstrated *in vivo* (11) using a resistant Guadeloupean stain of *B. glabrata* exhibiting allelic variation in a gene complex designated the Guadeloupe resistance complex (GRC) (12, 13). Notably, RNAi knockdown of a novel transmembrane protein encoded within the GRC appears to influence the resistance phenotype (no development of *S. mansoni*) (13) but the mechanism(s) by which this is accomplished remains unknown.

Although comparative transcriptomic analyses have been performed on whole snails or circulating hemocytes from different *B. glabrata* strains (14–17) and/or on snails in response to schistosome infection (18–22), evaluation of parasite-induced changes in hemocyte protein expression, specifically in hemocytes actively participating in sporocyst encapsulation reactions, have not been examined in detail (23). Targeted investigations of specific hemolymph proteins, such as Cu/Zn superoxide dismutase SOD1 (24), HSP 70 and 90 (25, 26), fibrinogen-related proteins (27, 28), thioester-containing proteins (29), MIF (30), biomphalysin protease (31), Toll-like receptor (TLR) (32), hemopoeitic factor granularin (33), and an unknown protein encoded by gene *Grctm6* (13) have demonstrated functional associations with snail immunity to schistosome infection. The diversity of protein mediators identified in these studies suggest that the molecular events transpiring prior to and during the hemocyte encapsulation reactions likely are complex, involving multiple protein components that differ depending on the specific snail-schistosome strain combination under investigation (3, 34, 35).

In the present study we performed a comparative proteomic analysis on hemocytes from inbred schistosome-susceptible (NMRI) and—resistant (BS-90) strains of *B. glabrata* during their active participation in *in vitro* encapsulation reactions with primary sporocysts of *S. mansoni* (NMRI strain). Specifically, we addressed the questions: What proteins are expressed by hemocytes directly participating in encapsulation reactions? Do susceptible and resistant snail hemocytes exhibit differential protein expression responses in the presence of encapsulated sporocysts? In order to address these questions we combined two powerful analytical methods, laser-capture microdissection (LCM) microscopy and nano-LC tandem mass spectrometry, to provide the first comparative proteomic analysis of schistosome-susceptible and—resistant hemocytes during active *in vitro* encapsulation of *S. mansoni* sporocysts.

## Materials and methods

### Ethics statement

Mice were maintained in an AAALAC-approved animal housing facility (Charmany Instructional Facility, University of Wisconsin-Madison) using standard-of-care cage housing and feeding. All animal handling and experimental protocols were approved by the University of Wisconsin-Madison Institutional Animal Care and Use Committee (IACUC) under Animal Welfare Assurance No. A3368-01.

### Maintenance of *B. glabrata* snails

Two inbred laboratory strains of *B. glabrata* snails, the *S. mansoni*-susceptible NMRI and -resistant BS-90 strains, were used in the present study. Snails were maintained in 20-gal aquaria at 26°C under a 12 h light:12 h dark photoperiod and fed leaf-lettuce *ad libitum* supplemented with Tetramin® fish food and chalk.

### Isolation of miracidia and sporocyst preparation

Mice, infected with the NMRI strain of *S. mansoni*, were provided by the Biomedical Research Institute (Rockville, MD) under a NIAID contract to BEI Resources (Manassas, VA). At 7 weeks post-infection, mice were euthanized and necropsied for removal of livers and axenic egg isolation (36).

Miracidia were isolated under axenic conditions as previously described (36, 37) and placed into *in vitro* culture in 24-well tissue culture plates (Corning Costar, NY) containing Chernin's balanced salt solution (CBSS) (38) under normoxic conditions at 26°C. The CBSS was supplemented with penicillin (100 U/mL), streptomycin sulfate (0.1 mg/mL) and 1 g/L each of glucose and trehalose. Following miracidial transformation (24 h), primary sporocysts were maintained in culture for an additional 24 h at 26°C, after which they were washed 5 times in CBSS by gravity sedimentation to remove the shed epidermal plates and other cellular debris.

### Hemocyte preparation

Hemolymph (HL) from 120 *B. glabrata* snails (12–15 mm shell diameter) of each strain was removed by the headfoot retraction method (39). Briefly, prior to bleeding, snail shells were wiped with cotton swabs soaked in 70% ethanol and air dried, followed by carefully peeling back the shell to expose the headfoot region. A rapid poking of the tissue by forceps forced the headfoot to retract while extruding HL from the hemal pore. The HL was then collected by micropipettor using siliconized, sterilized tips, and dispensed onto a cold sterile glass plate for 15–20 s to allow for remove any shell debris or mucus before transfer to a sterile 15-mL conical centrifuge tube containing cold CBSS. The tube was kept on ice during HL collection, with the final HL: CBSS ratio after collection equaling ~1:1. All procedures outlined above for HL extraction were performed in a biosafety cabinet to minimize microbial contamination. Hemocyte concentrations of pooled samples were determined by Neubauer hemocytometer (318 ± 131 and 304 ± 56 cells/μL in BS-90 and NMRI, respectively).

### Hemocyte-sporocyst cell-mediated cytotoxicity (CMC) assay

The CMC assay was modified from that reported by Bayne et al. (8) and Hahn et al. (40). After adjusting cell concentrations for NMRI and BS-90 hemocyte preparations, HL was transferred to 1.5 mL siliconized, sterile microcentrifuge tubes containing approximately 100 *in vitro* transformed *S. mansoni* primary sporocysts. Each tube contained a cushion of 0.5% agarose dissolved in CBSS at its bottom to provide a substrate for hemocytes and sporocysts to interact. Tubes were centrifuged at 1,000 rpm (Eppendorf 5415D; Brinkmann Instruments, Westbury, NY) for 10 min to bring hemocytes into contact with the sporocysts at the bottom of tubes. The control groups consisted of introducing pooled HL to identical tubes containing agarose plugs, but in the absence of sporocysts. In this case, hemocyte aggregates, resembling larval encapsulations, spontaneously formed at the agarose surface. These were processed identically to larval-containing cell preparations.

### Sample collection and cryosectioning

**Figure 1:**
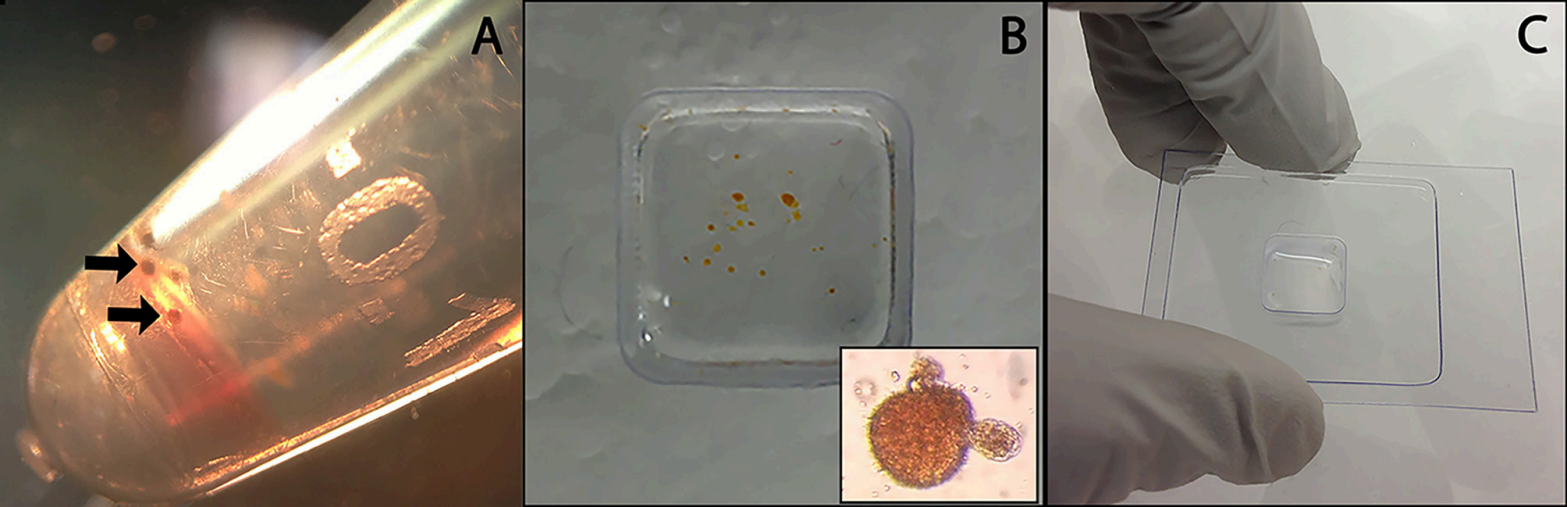
Preparation of control *B. glabrata* hemocyte aggregates and hemocyte-encapsulated *S. mansoni* sporocysts for cryohistology. Hemocyte-sporocyst encapsulations (arrows) following larval-hemolymph coculture for 18 h and washing in CBSS **(A)**; Isolated hemocyte samples placed in a cryo-mold (insert hemocyte-sporocyst aggregate) **(B)**; Quick freezing of cellular encapsulations in OTC prior to cryosectioning **(C)**.

Hemocyte/sporocyst and hemocyte only cultures were incubated undisturbed for 18 h at 26°C under normoxic conditions. Following incubation, cell clumps (Figure 1A) were washed 5x with CBSS, transferred to cryomolds, and allowed to settled (Figure 1B). CBSS was then removed and replaced by the cryosectioning embedding medium, OCT (Lab-Tek Products, Naperville, IL) (Figure 1C), followed immediately by freezing on dry ice and storage at −80°C until sectioning. Frozen sections (10 μm thickness) of OTC-embedded NMRI and BS-90 hemocyte aggregates were cut using a Leica CM 1900 cryostat (Leica) at −28°C, placed on membrane-coated glass slides (Molecular Machines & Industries-MMI microslides, Haslett, MI) and maintained at −80°C before LCM. Slide-mounted cryosections were fixed in 70% EtOH for 30 sec, stained with Mayer's hematoxylin solution (30 s) (Sigma-Aldrich), wash 2x in Milli-Q water (15 s), and dehydrated in a graded EtOH series consisting of 70% (10 s), 2 × 95% (10 s), and 2 × 100% (10 and 30 s) absolute EtOH. The stained tissue sections were air-dried in a laminar flow hood for 15 min and then subjected to LCM.

### Laser-capture microdissection (LCM)

**Figure 2:**
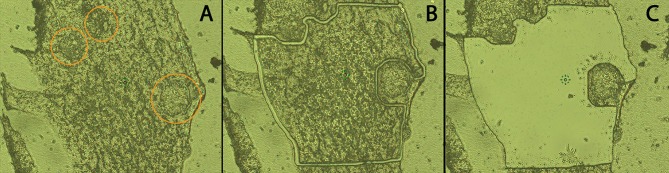
Cryosection of *in vitro* hemocyte encapsulation containing *S. mansoni* sporocysts. Following location of encapsulated sporocysts (**A**; circled), hemocyte and sporocyst tissue sections were dissected by laser beam using LCM **(B)**, and hemocyte tissue subsequently transferred to ISOLATION-CAP™ tubes **(C)** for protease digestion and MS processing (20x).

LCM was used to separate and isolate hemocytes (Hc) from encapsulated sporocysts in order to enrich for Hc-associated proteins in Hc-sporocyst co-cultivation samples. The MMI CellCut® Nikon microscope (Molecular Machines & Industries, Haslett, MI) was used to image and microdissect encapsulating tissue. Using the MMI CellCut® software, regions of interest were selected in the tissue images (Figure 2A) and microdissected with a laser (Figures 2B,C). The dissected tissue sections were then collected in MMI IsolationCap® tubes for subsequent proteomic analysis. Approximately 3 × 10^6^ μm^2^ of sectioned tissue per sample were collected with one experiment consisting of four sample collections: BS-90 Hc only (BS90), BS-90 Hc + sporocysts (BS90-SP), NMRI Hc only (NMRI), NMRI Hc + sporocysts (NMRI-SP). Two independent experiments were conducted, each consisting of fresh pooled hemolymph and a new batch of transformed sporocysts.

### Proteomic analysis

Sectioned microdissected tissue in microcap tubes were solubilized and protein denatured in 12 μL of a chaotropic detergent containing solution [6M Urea/0.5% ProteaseMAX™ surfactant (Promega Corp., Madison, WI) in 50 mM NH_4_HCO_3_ (pH 8.5)] for 1 min at 24°C then switched to 42°C for 10 min and subsequently diluted to 60 μL in a reduction step by addition of 2.5 μL of 25 mM dithiothreitol (DTT), 5 μL MeOH and 40.5 μL of 25 mM NH_4_HCO_3_ (pH 8.5). Samples were then incubated at 52°C for 15 min, cooled on ice to 22°C, followed by addition of 3 μL of 55 mM iodoacetamide for alkylation in the dark for 15 min at 22°C. Reactions were quenched by adding 6 μL of 25 mM DTT. Protease digestion was initiated by adding 4 μL of Trypsin/LysC solution [10 ng/μL Trypsin/LysC mix (Promega) in 25 mM NH_4_HCO_3_] and 27 μL of 25 mM NH_4_HCO_3_ (pH 8.5) to each sample tube for 2 h at 42°C, followed by the addition of 3 μL more of Trypsin/LysC solution and continued overnight digestion at 37°C. Reactions were terminated by acidification with 2.5% trifluoroacetic acid (TFA, Sigma-Aldrich) to 0.3% final concentration.

Digests were cleaned up using OMIX C18 SPE cartridges (Agilent Technologies, Palo Alto, CA) per the manufacturer's protocol and eluted in 10 μL of 70/30/0.1% acetonitrile (ACN)/H_2_O/TFA, dried to completion in a speed-vac centrifuge and finally reconstituted to 20 μL in 0.1% formic acid. Peptides were analyzed by nanoLC-MS/MS using an Agilent 1100 nanoflow system connected to a hybrid linear ion trap-orbitrap mass spectrometer (LTQ-Orbitrap XL, Thermo Fisher Scientific) equipped with a nanoelectrospray ion source. Chromatography of peptides prior to mass spectral analysis was accomplished using a C18 reverse-phase HPLC trap column (Zorbax 300SB-C18, 5 μM, 5 × 0.3 mm, Agilent Technologies) and capillary emitter column (in-house packed with MAGIC C18, 3-μM, 150 × 0.075 mm; Michrom Bioresources, Inc., Sacramento, CA) onto which 8 μL of digest was automatically loaded. A nanoHPLC system delivered solvents A (0.1% (v/v) formic acid) and B (95% (v/v) ACN, 0.1% (v/v) formic acid) at a rate of 10 μL/min to load the peptides (over 45 min) and 0.2 μL/min to elute peptides directly into the nano-electrospray with a continuous gradient from 0% (v/v) B to 40% (v/v) B over the next 145 min. This was followed by a fast gradient elution from 40% (v/v) B to 60% (v/v) B for 20 min and finally a 5 min flash-out from 50 to 95% (v/v) B. As peptides eluted from the HPLC-column/electrospray source, survey MS scans were acquired in the Orbitrap with a resolution of 100,000, and up to 5 most intense peptides per scan were fragmented and detected in the ion trap over the 300–2,000 m/z, with redundancy being limited by dynamic exclusion.

### Bioinformatic analyses

Raw MS/MS data were converted to mgf file format using the Trans-Proteomic Pipeline (http://tools.proteomecenter.org/software.php; Seattle Proteome Center, Seattle, WA). Resulting mgf files were used to search against concatenated *B. glabrata* and *S. mansoni* protein databases consisting of RefSeq non-redundant entries plus common lab contaminants and decoy entries for false discover rate (FDR) calculations (96,790 total entries) with cysteine carbamidomethylation as fixed modification plus methionine oxidation and asparagine/glutamine deamidation as variable modifications. Peptide mass tolerance was set at 15 ppm and fragment mass at 0.6 Da. Thermo Proteome Discoverer version 1.4.1.14 was used to integrate results from dual search engines (Sequest and Mascot) and Scaffold (version Scaffold_4.3.2, Proteome Software Inc., Portland, OR) was used to validate MS/MS-based peptide and protein identifications. Peptide identifications were accepted if they could be established at >84.0% probability to achieve an FDR < 1.0% by the Scaffold Local FDR algorithm. Protein identifications were accepted if they achieved an FDR < 1.0% and contained at least 2 identified peptides. Protein probabilities were assigned by the Protein Prophet algorithm (41). Proteins that contained similar peptides and could not be differentiated based on MS/MS analysis alone were grouped to satisfy the principles of parsimony. The complete raw dataset used in bioinformatics analyses of the hemocyte proteomic data are available as project #1492 available at chorusproject.org SMART (Simple Modular Architecture Research Tool) was employed to identify protein domains using normal mode, including outlier homologs and PFAM domains (http://smart.embl-heidelberg.de/smart/set_mode.cgi?NORMAL=1). Clustal Omega (http://www.ebi.ac.uk/Tools/msa/clustalo/) (42) was used for multiple-sequence alignments. Unknown/uncharacterized/hypothetical proteins were subjected to BLASTp searches of the nrNCBI db (as of May 2018) to acquire related protein identifications. The entire experiment was performed twice on different dates using different pools of hemolymph and *in vitro* cultured sporocysts. The results generated in both experiments were combined into a single dataset to provide an overall assessment of the proteomic response of encapsulating hemocyte in the presence and absence of larval *S. mansoni*. The combined dataset from each snail strains was then subjected to pair-wise comparison (NMRI vs. NMRI-SP, BS-90 vs. BS-90-SP, and BS-90-SP vs. NMRI-SP) of unique and commonly-occurring proteins identified in each combination. All proteins identified as *S. mansoni* were removed from the analyses. Unique proteins (identified in only one of the pair) were further trimmed by removing all proteins with a total normalized spectral counts of <2. The remaining unique proteins were then identified and gene ontology analyses were performed using Uniprot. Similarly, proteins shared in common in each paired comparison were further processed by removing proteins with total normalized spectral counts of <4. In anticipation of analyzing these common proteins for potential differential expression, we verified that each experiment had comparable protein sample sizes using normalized spectral counts of actin (XP_013070997.1, XP_013083901.1, XP_013083930.1, XP_013083929.1) as a housekeeping protein. By comparing the average actin spectral counts identified within each proteomic database, we observed count differences ranging from 1.2 to 18% between paired samples (BS-90 vs. BS-90-SP: 18%; NMRI vs. NMRI-SP: 1.2%; and BS-90-SP vs. NMRI-SP: 7%). Given this range of sampling, for proteins shared in common between groups, a fourfold difference in total normalized spectral counts was imposed as a conservative cut-off for determining differential protein expression in pairwise treatment comparisons.

## Results and discussion

In an effort to better understand the molecular events associated with the differences observed at the host-parasite interface of *B. glabrata* and *S. mansoni*, a proteomic analysis of hemocytes actively engaged in the encapsulation of *S. mansoni* primary sporocyst was conducted. A MALDI-MS/MS approach was undertaken to analyze the expressed protein profile of hemocytes of inbred resistant BS-90 and susceptible NMRI *B. glabrata* snail strains involved in the encapsulation of *S. mansoni* (NMRI strain) primary sporocyst. The main goal of the study was to show the differential expression pattern of proteins expressed in hemocytes during the encapsulation of *S. mansoni* sporocyst, and to compare the proteins expressed in hemocytes of these snail strains in order to provide a better understanding of the molecular events associated with the innate immune response of *B. glabrata* snails to early developing sporocysts. Using this comparative proteomics approach, a total of 1061 *B. glabrata* hemocyte proteins were identified from cellular capsules in the presence and/or absence (control) of *in vitro* co-cultured *S. mansoni* sporocysts. Although an additional 155 *S. mansoni* proteins were identified, the current study will focus on hemocyte responses during parasitic encapsulation. All identified *B. glabrata* and *S. mansoni* proteins are listed in Supplementary Table 1 and include normalized spectral counts per protein for each treatment. Raw MS proteomics data can be downloaded at the website address:

https://chorusproject.org/anonymous/download/experiment/defa50bf549a41dda7a156928ca0a03f under project reference number 1492.

### Proteomic response of hemocytes to encapsulated sporocysts

**Figure 3:**
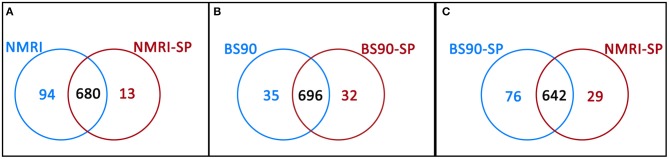
Venn diagrams illustrating the number of unique proteins and proteins shared in common between **(A)** NMRI control (NMRI) and NMRI-sporocyst (NMRI-SP) encapsulations; **(B)** BS-90 control (BS90) and BS-90-sporocyst (BS90-SP) encapsulations and **(C)** sporocyst encapsulations by NMRI and BS-90 *B. glabrata* hemocytes (NMRI-SP and BS90-SP).

Hemocytes of both the NMRI and BS-90 *B. glabrata* strains readily encapsulated *S. mansoni* sporocysts (SP) *in vitro* forming cellular aggregation of various sizes (Figures 1A,B). In the absence of sporocysts (controls), hemocytes of both snail strains also spontaneously formed cellular capsules of similar size and number as corresponding co-cultures. This design permitted simultaneous comparisons of expressed hemocyte proteins between snail strains in the presence and absence of sporocysts. As shown in Venn diagrams (Figure 3), following application of filtering criteria, the numbers of unique and common hemocyte proteins identified in control/sporocyst (NMRI/NMRI-SP, BS90/BS90-SP) and sporocyst/sporocyst (NMRI-SP/BS90-SP) combinations for each snail strain are presented. Table 1 highlights a list of selected identified unique proteins of particular interest in this study and are designated as presence (+), absence (−) or presence, but below the defined threshold cutoff (+/−) for each host/parasite comparison listed above. Similarly, proteins found to be shared in common among all compared treatments and strains, and presenting with a 4-fold difference in total normalized spectral counts, are listed in Tables 2–4.

**Table 1:** Unique hemocyte proteins of interest identified in paired comparisons of susceptible (NMRI) and resistant (BS90) strains of *B. glabrata* in the presence and absence of *S. mansoni* sporocysts (SP) (NMRI vs. NMRI-SP; BS90 vs. BS90-SP; NMRI-SP vs. BS90-SP).

**UNIQUE proteins**		**NMRI**	**NMRI-SP**	**BS90**	**BS90-SP**	**NMRI-SP**	**BS90-SP**
**NCBI acc. no**.	**Protein identification**						
**IMMUNE RELATED**							
XP_013062219.1	Integrin alpha-PS1-like [Bg]	+	−	−	−	−	−
XP_013064668.1	*/Fibrinogen-related protein F precursor [Bg] AQX34583.1 (96%)	+	−	+	+/−	−	+/−
XP_013070670.1	lipopolysaccharide-binding protein-like isoform X1 [Bg]	−	−	+/−	+	−	+
XP_013064836.1	*/ Interleukin-1 receptor-associated kinase -a [*Mytilus galloprovincialis*] AHI17298.1 (31%)	−	+	−	−	+	−
XP_013089248.1	Piwi-like protein 1 [Bg]	+	−	+	−	−	−
XP_013068459.1	Protein argonaute-2-like isoform X1 [Bg]	−	−	+	−	−	−
XP_013079233.1	Protein argonaute 18-like [Bg]	−	+	−	+/−	+	+/−
XP_013060966.1	Histone H2A.V [Bg]	−	+	+	+	+	+
XP_013088376.1	Bactericidal permeability-increasing protein-like [Bg]	+/−	+	−	+	+	+
XP_013095937.1	Bactericidal permeability-increasing protein-like [Bg]	+/−	−	+/−	+	−	+
XP_013064309.1	Fibrinogen-like protein A [Bg]	−	−	+	+	−	+
XP_013065165.1	*/ CD209 antigen-like protein 2 [Bg] XP_013095847.1 (56%)	+	+	+/−	−	+	−
XP_013064709.1	*/Pannexin10 [*Aplysia californica*] NP_001191462.1 (24%)	+/−	+	−	−	+	−
XP_013075978.1	Perilipin-2-like [Bg]	+	−	+/−	+	−	+
XP_013069899.1	GRB2-associated-binding protein 1-like isoform X1 [Bg]	+	−	−	−	−	−
XP_013077735.1	Alpha-L-fucosidase-like [Bg]	+	−	−	−	−	−
XP_013066054.1	Sequestosome-1-like [Bg]	+	−	+	+/−	−	+/−
XP_013067929.1	Leucine-rich repeat-containing protein 47-like [Bg]	+	−	−	−	−	−
**REDOX**							
XP_013077425.1	Ferric-chelate reductase 1 [Bg]	+	−	+	+/−	−	+/−
XP_013084576.1	Glutaredoxin-3-like [Bg]	+	−	−	+/−	−	+/−
XP_013091598.1	Peroxidasin homolog [Bg]	+	−	−	−	−	−
XP_013077074.1	SH3 and PX domain-containing protein 2A-like isoform X1 [Bg]	+	+	−	−	+	−
XP_013076408.1	Glutarate-semialdehyde dehydrogenase DavD-like, partial [Bg]	+	+	+	−	+	−
XP_013068651.1	Microsomal glutathione S-transferase 3-like [Bg]	+	+/−	+	−	+/−	−
XP_013091634.1	D-arabinono-1,4-lactone oxidase-like [Bg]	+	+	−	+	+	+
XP_013070344.1	Superoxide dismutase [Cu-Zn]-like [Bg]	+/−	−	−	+	−	+
XP_013084558.1	Dual oxidase-like [Bg]	−	−	+/−	+	−	+
XP_013060671.1	Glutathione S-transferase 4-like [Bg]	+/−	−	+	+	−	+
XP_013090296.1	Nicotinamide phosphoribosyltransferase-lie, partial, [Bg]	−	−	−	+	−	+
XP_013085832.1	Laccase-3-like isoform X1 [Bg]	+/−	−	−	+	−	+
XP_013073837.1	Isocitrate dehydrogenase [NADP] cytoplasmic-like isoform X1 [Bg]	+	+	+	−	+	−
XP_013083111.1	NADPH−cytochrome P450 reductase-like [Bg]	+	−	+/−	+	−	+
NP_976169.1	Cytochrome c oxidase subunit II (mitochondrion) [Bg]	+	+	+	−	+	−
XP_013067188.1	Cytochrome P450 2J3-like [Bg]	−	−	+	+	−	+
**APOPTOSIS**							
XP_013071242.1	*/ E3 ubiquitin-protein ligase MIB1-like [Bg]; XP_013087470.1 (51%)	−	+/−	+	−	+/−	−
XP_013086341.1	Death-associated protein 1-like [Bg]	−	−	−	+	−	+
XP_013065969.1	Bcl-2-related protein A1-like [Bg]	+	−	+	+	−	+
XP_013094855.1	Ubiquitin-like modifier-activating enzyme 6 isoform X1 [Bg]	+	+/−	−	+	+/−	+
XP_013088836.1	TAR DNA-binding protein 43-like [Bg]	−	+	+	−	+	−
**STRESS RESPONSE**							
XP_013064401.1	T-complex protein 1 subunit gamma-like, partial [Bg]	+	−	−	+	−	+
XP_013064658.1	Hsc70-interacting protein-like [Bg]	−	−	+/−	+	−	+
XP_013061307.1	Prefoldin subunit 1-like [Bg]	+	+	−	−	+	−
XP_013068854.1	Parkin coregulated gene protein homolog isoform X1 [Bg]	−	+	−	−	+	−
XP_013077814.1	CDGSH iron-sulfur domain-containing protein 2-like [Bg]	−	−	−	+	−	+
XP_013094352.1	Serine/threonine-protein kinase fray2-like [Bg]	−	+	−	−	+	−
**SIGNALING**							
XP_013077238.1	Coronin-1A-like [Bg]	+	+	+	−	+	−
XP_013070025.1	Rap1 GTPase-activating protein 1-like isoform X4 [Bg]	+	−	+/−	−	−	−
XP_013074990.1	COP9 signalosome complex subunit 6-like [Bg]	+	−	+/−	−	−	−
XP_013086090.1	COP9 signalosome complex subunit 4-like isoform X1 [Bg]	+	−	+	+	−	+
XP_013078600.1	SH3 domain-containing kinase-binding protein 1-like isoform X1 [Bg]	+	−	+/−	+/−	−	+/−
XP_013087123.1	SHC-transforming protein 1-like [Bg]	−	−	+	−	−	−
XP_013088835.1	*/SH3 Domain-containing guanine exchange factor [*Crassostrea gigas*]; EKC39783.1 (48%)	+	−	+/−	+	−	+
XP_013087149.1	*/Tensin-1-like, partial [*Aplysia californica*] (49%)	+	−	+/−	+/−	−	+/−
XP_013078554.1	*/Receptor-type tyrosine-protein phosphatase T-like isoform X1 [Bg]; XP_013072072.1 (40%)	+	−	+	+	−	+
**CALCIUM BINDING**							
XP_013077729.1	Calmodulin-like, partial [Bg]	−	−	+	+	−	+
XP_013079182.1	*/Microtubule-actin cross-linking factor 1-like isoform X13 [Bg]; XP_013079259.1 (99%)	+/−	−	+	−	−	−
XP_013083369.1	Clathrin interactor 1-like isoform X3 [Bg]	+	+	−	−	+	−
XP_013091491.1	16 kDa calcium-binding protein-like [Bg]	−	−	+/−	+	−	+
XP_013072348.1	Calcineurin subunit B type 1 [Bg]	−	−	−	+	−	+
XP_013060775.1	Calumenin-like [Bg]	−	−	+/−	+	−	+
XP_013066201.1	Gelsolin-like protein 1, partial [Bg]	−	−	+/−	+	−	+
XP_013068775.1	Gelsolin-like protein 1 [Bg]	+	−	−	+	−	+
XP_013088705.1	Peflin-like [Bg]	+	+	+	−	+	−
XP_013086544.1	Nucleobindin-2-like [Bg]	+/−	+/−	−	+	+/−	+
**TRANSCRIPTION/TRANSLATION PROCESS**							
XP_013063581.1	60S Ribosomal protein L14-like [Bg]	+/−	−	+	+	−	+
XP_013075095.1	60S Ribosomal protein L31-like [Bg]	+	−	+	+	−	+
XP_013089238.1	60S Ribosomal protein L36-like [Bg]	+	−	+	+/−	−	+/−
XP_013096652.1	60S Ribosomal protein L37a-like [Bg]	+	−	+	+	−	+
XP_013091387.1	Host cell factor-like isoform X1 [Bg]	+	−	+	−	−	−
XP_013062003.1	LIM and senescent cell antigen-like-containing domain protein1 isoform X1 [Bg]	−	+/−	−	+	+/−	+
XP_013062476.1	Signal recognition particle subunit SRP72-like [Bg]	+	−	+	+	−	+
XP_013093252.1	*/Histone-lysine N-methyltransferase MLL4 [*Crassostrea gigas*]; EKC19620.1 (61%)	+	+/−	+	−	+/−	−
XP_013072871.1	Krueppel homolog 1-like isoform X1 [Bg]	+	−	−	−	−	−
**METABOLISM**							
XP_013083732.1	Inter-alpha-trypsin inhibitor heavy chain H3-like isoform X1 [Bg]	+	+	−	−	+	−
XP_013079110.1	Alpha-N-acetylglucosaminidase-like isoform X1 [Bg]	+	−	+	+/−	−	+/−
XP_013080405.1	Gamma-glutamyl hydrolase A-like [Bg]	−	−	−	+	−	+
**TRAFFICKING**							
XP_013083454.1	Intersectin-1-like [Bg]	+	−	+/−	−	−	−
XP_013094430.1	Nuclear pore membrane glycoprotein 210-like [Bg]	+/−	−	+	−	−	−
XP_013083696.1	AP-3 complex subunit delta-1-like isoform X1 [Bg]	+	−	+/−	+/−	−	+/−
XP_013060735.1	Sorting nexin-33-like, partial [Bg]	+	−	+	+/−	−	+/−
**MISC**.							
XP_013092372.1	Endothelin-converting enzyme 2-like [Bg]	−	−	+	−	−	−
XP_013063554.1	Basement membrane-specific heparan sulfate proteoglycan core protein-like [Bg]	+	+	+	−	+	−

Unique proteins are organized by functional catagory and NCBI accession numbers and protein identification are listed. For each paired comparison, (+) indicates the presence of the protein; (−) indicates the absence of the protein and (+/−) indicates the protein presence, but the threshold of less than 2 peptides was not met. Uncharacterized protein are indicated (*/), followed by their closest NCBI Blastp IDs and ID percentages.

**Table 2:** Proteins shared in common between control NMRI and sporocyst-encapsulating (NMRI-SP) hemocytes that present at least a 4-fold difference in total normalized spectral counts.

**NMRI vs. NMRI-SP: 4 fold difference**				
**NCBI acc. no**.	**Protein identification**	**NMRI**	**NMRI-SP**	**Function**
**IMMUNE RELATED**				
XP_013070669.1	Lipopolysaccharide-binding protein-like [Bg]	9.57	1.01	Cellular defense
XP_013070436.1	Syntenin-1-like isoform X1 [Bg]	11.98	0.96	Immunomodulation
XP_013082155.1	Apolipophorins-like isoform X1 [Bg]	9.57	0.96	Detox LPS
XP_013067749.1	Lysosomal alpha-mannosidase-like, partial [Bg]	5.30	0.96	Killing of foreign material
XP_013066053.1	*/ Sequestosome-1-like [*Limulus polyphemus*] XP_013792674.1 (36%)	18.78	0.96	Autophagy
XP_013093397.1	Lysosomal alpha-glucosidase-like isoform X1 [Bg]	7.44	1.01	Degradation of Phagocytosed materials
XP_013088376.1	Bactericidal permeability-increasing protein-like [Bg]	1.11	4.89	Anti-microbial
XP_013074089.1	Arginase-1-like isoform X3 [Bg]	15.05	65.36	Immune response
**REDOX**				
XP_013077724.1	*/ Carbonyl reductase [NADPH] 1-like [Bg] XP_013077725.1 (74%)	4.27	0.96	Redox
XP_013076465.1	3-Oxo-5-alpha-steroid 4-dehydrogenase 1-like [Bg]	4.37	1.01	NADPH binding/Redox
XP_013093038.1	Succinate dehydrogenase [ubiquinone] iron-sulfur subunit, mitochondrial-like [Bg]	1.02	6.86	Mitochondrial electron transport
XP_013073195.1	Cytochrome c oxidase subunit 4 isoform 1, mitochondrial-like [Bg]	1.11	4.84	Mitochondrial electron transport
**STRESS RESPONSE**				
XP_013091012.1	Stromal cell-derived factor 2-like isoform X1 [Bg]	4.27	1.01	Regulation apoptosis/ chaperonin
XP_013065387.1	Protein FAM107B-like, partial [Bg]	4.27	0.96	Similarity with heat-shock proteins
**SIGNALING**				
XP_013088940.1	G-protein coupled receptor 64-like isoform X1 [Bg]	4.18	0.96	Signaling proteins
XP_013077039.1	*/ Receptor-type tyrosine-protein phosphatase α-like [Bg] XP_013063115.1 (42%)	4.27	0.96	MAPK cascade
XP_013077041.1	Receptor-type tyrosine-protein phosphatase alpha-like, partial [Bg]	5.30	0.96	MAPK cascade
XP_013081619.1	Serine/threonine-protein kinase PAK 2-like [Bg]	8.55	1.97	Signaling
**CELL ADHESION**				
XP_013081823.1	Collagen alpha-1(XVIII) chain-like [Bg]	8.55	1.91	Cell adhesion
XP_013086335.1	*/ Neurexin-1-like [Aplysia californica] XP_012941838.1 (48%)	38.20	1.91	Cell adhesion
**TRANSCRIPTION/TRANSLATION PROCESS**				
XP_013061577.1	40S Ribosomal protein S25-like [Bg]	4.27	0.96	Ribosomal
XP_013084756.1	60S Ribosomal protein L19-like, partial [Bg]	4.18	1.01	Ribosomal
XP_013071682.1	60S Ribosomal protein L30-like [Bg]	4.37	0.96	Ribosomal
XP_013073467.1	60S Ribosomal protein L34-like [Bg]	5.39	1.01	Ribosomal
XP_013063478.1	60S Ribosomal protein L8 [Bg]	21.55	4.95	Ribosomal
XP_013084934.1	Heterogeneous nuclear ribonucleoprotein U-like protein 1 isoform X1 [Bg]	8.28	1.97	Transcription regulation
XP_013070062.1	Protein LZIC-like [Bg]	5.39	1.01	B-catenin binding
XP_013081352.1	Protein TFG-like isoform X1 [Bg]	4.37	0.96	Regulation of NFKB signaling
XP_013063363.1	Ribonuclease UK114-like [Bg]	5.30	1.01	Translation inhibition
XP_013062948.1	RNA-binding protein lark-like [Bg]	5.39	0.96	Translation regulation
XP_013075272.1	Serine/arginine repetitive matrix protein 2-like [Bg]	4.37	0.96	Pre-mRNA splicing
XP_013089331.1	Serine/arginine-rich splicing factor 7-like [Bg]	4.18	1.01	Pre-mRNA splicing
**METABOLISM PROCESS**				
XP_013069940.1	Alpha-galactosidase A-like isoform X1 [Bg]	5.48	1.01	Glycosidase
XP_013076662.1	Succinyl-CoA:3-ketoacid coenz. A transferase 1, mitochondrial-like [Bg]	8.37	1.01	Ketone body catabolism
XP_013062675.1	UTP–glucose-1-phosphate uridylyltransferase-like [Bg]	9.75	0.96	Cellular metabolic pathways
XP_013061266.1	1,4-alpha-glucan-branching enzyme-like [Bg]	4.27	0.96	Glycogen biosynthesis
XP_013092915.1	2-amino-3-ketobutyrate coenzyme A ligase, mitochondrial-like [Bg]	8.64	1.91	Cellular amino acid metabolism
XP_013094977.1	3-oxoacyl-[acyl-carrier-protein] reductase FabG-like [Bg]	5.48	1.01	Fatty acid biosynthesis
**PROTEIN SORTING AND TRAFFICKING**				
XP_013089878.1	AP-1 complex subunit beta-1-like [Bg]	9.85	1.91	Clathrin binding/ transporter activity
XP_013095772.1	Charged multivesicular body protein 4b-like [Bg]	12.73	2.92	Multivesicular body pathway
XP_013061300.1	FK506-binding protein 15-like [Bg]	5.39	1.01	Transport of early endosomes
XP_013067947.1	Huntingtin-interacting protein 1-like isoform X1 [Bg]	4.18	0.96	Trafficking
XP_013082360.1	Vacuolar protein sorting-associated protein 27 isoform X1 [Bg]	5.30	0.96	Trafficking
XP_013080513.1	Vacuolar protein sorting-associated protein 35-like [Bg]	6.50	0.96	Trafficking
**MISC**				
XP_013080947.1	*/Coiled-coil protein 90B, mitochondrial [*Crassostrea gigas*] EKC25784.1 (50%)	6.41	0.96	Transmembrane protein
XP_013080174.1	*/voltage-dependent calcium channel γ2 subunit-like [Bg] XP_013080170.1 (40%)	5.39	1.01	Voltage-dependent calcium channel
XP_013069538.1	V-type proton ATPase 116 kDa subunit a isoform 1 [Bg]	4.27	0.96	Proton transporter

NCBI accession numbers and protein identification are listed. Color backgrounds indicate an increased spectral count for proteins in the corresponding treatment. Uncharacterized proteins (*/) are followed by their closest NCBI Blastp IDs and ID percentages.

### Proteomic response of susceptible (NMRI) snail hemocytes to encapsulated sporocysts

In the susceptible NMRI strain of *B. glabrata*, 680 hemocyte proteins were shared between the control and sporocyst-encapsulated groups, while 94 proteins were unique to the NMRI-control group and 13 to the NMRI-SP group (Figure 3A). Of the hemocyte proteins shared in common, 47 showed at least a 4-fold difference between control and sporocyst encapsulations. Of these 47 common proteins, the vast majority (43/47; 91%) exhibited a striking reduction in specific proteins in the presence of sporocysts, suggesting a downregulation of expression in susceptible snail cells in response to parasite exposure (Table 2). This parasite-associated decrease in NMRI hemocyte protein expression is also mirrored in the number of unique proteins identified in control (94 proteins) compared to sporocyst (13 proteins) encapsulations.

The majority of proteins (4-fold and unique) observed to be down-regulated in presence of parasites appears to involve protein synthesis/trafficking, metabolism, cell signaling, or oxidation-reduction (redox) activities (Table 2), all of which may have implications for an impaired immune capability. Among the down-regulated NMRI hemocyte proteins identified as potentially involved in immune-related functions in the presence of encapsulated sporocysts include the following: LPS binding protein (LBP), an antimicrobial protein capable of interacting with the larval tegument and secretions (43); apolipophorin, an immune-stimulating molecule in insects (44), found to be downregulated in snails exposed to microbial antigens (LPS, FCN, PGN) (45); and sequestosome 1/p62, a protein involved in various mechanisms, including apoptosis, innate immunity and autophagy (46). In addition to binding ubiquinated proteins for basal cellular autophagy, p62 may also target intracellular pathogens for degradation through a p62-dependent selective autophagy mechanisms and participate in host defense. For example, in murine macrophages, activation of this selective autophagic mechanism by LPS-triggered TLR4 requires upregulation of p62 expression, suggesting a role of p62 in innate immune responses to pathogens (47). Of relevance here, TLRs have been implicated in *B. glabrata-S. mansoni* interactions as demonstrated by a differential increase in *Bg*TLR protein levels associated with snail strains exhibiting resistance to *S. mansoni* (32). Our finding that p62/sequestosome1 is dramatically decreased in NMRI-SP capsules, suggests an impairment of hemocyte p62-mediated selective autophagy by a parasite-induced targeting of p62. A similar disruption of p62 has been postulated as a survival mechanism by some pathogens (46).

By contrast, 4 immune-related NMRI hemocyte proteins exhibited an increase in expression in the presence of parasites: An uncharacterized protein, containing a N-terminal death domain of interleukin-1 receptor-associated kinases (IRAK) followed by a catalytic domain of the serine/threonine kinases, argonaute 18, bactericidal permeability increasing protein (BPI) and arginase 1. Although uncharacterized, the presence of the death-domain of IRAK and serine/threonine kinase suggests this protein belongs to the IRAK superfamily, with a potential involvement in the Toll pathway (48). The presence of argonaute 18 suggests the involvement of small RNAs, possibly in gene expression regulation via RISC (RNA-induced silencing complex). *B. glabrata* snails (Belo Horizonte strain) exposed to *S. mansoni* exhibited an upregulation of argonaute gene expression at 24 h post-infection, consistent with our current proteomic data (49). Our observed increase in BPI, a pattern recognition protein known to bind to lipopolysaccharides of Gram-negative bacteria (50), in hemocytes of both NMRI and BS-90 snails participating in larval encapsulations is in contrast to a reported decrease in BPI transcript response to the echinostome *Echinostoma caproni* in resistant *B. glabrata* (51), suggesting a potential pathogen specificity for BPI expression between *S. mansoni*-resistant *B. glabrata* strains. Finally, the finding that arginase1 expression is highly increased in the presence of sporocysts in susceptible host capsules may be of functional significance. Arginase is known to inhibit the production of NO by directly competing with nitric oxide synthase (NOS) for L-arginine (substrate), resulting in reduced levels of larval-killing NO production (52). Therefore, a parasite-induced triggering of hemocyte arginase production, as shown in the present study, may be serving as an anti-immune survival mechanism by creating a detoxified cellular microenvironment for encapsulated sporocysts.

Although proteins involved in redox reactions were found to be mostly down-regulated in presence of parasites (3-oxo-5-alpha-steroid 4-dehydrogenase 1; NADPH-cytochrome P450 reductase; peroxidasin; glutaredoxin-3; ferric-chelate reductase 1), two others, cytochrome c oxidase (Cyt c) and succinate dehydrogenase (ubiquinone-containing complex) exhibited increased protein expression. Cyt c and ubiquinone are part of the mitochondrial electron transport chain that, in addition to generating ATP, also are involved in the production of ROS. However, it is not known whether metabolically-generated ROS from this source may be serving a host immune effector role. More likely, the increased expression of these proteins probably reflects an enhancement of metabolic stress of host cells in response to sporocysts.

Overall, hemocytes of susceptible NMRI snails in the presence of *S. mansoni* sporocysts appear to have 2 distinct responses: A general down-regulation of proteomic activity across various protein groups, and a discrete increase of selected proteins, suggesting that different mechanisms potentially may be initiated either by the host or parasite. Although ROS, BPI and the death domain-IRAK containing protein increases are likely to be initiated by the host in response to a pathogen invasion, the increase of arginase, and with it, a resulting decrease in toxic NO production, or the general protein knockdown observed in NMRI hemocytes, could be a direct result of host modulation by the parasite to favor its own survival.

### Proteomic response of resistant (BS-90) snail hemocytes to encapsulated sporocysts

As a result of proteomic analysis of BS-90 hemocyte capsules, 696 proteins were identified as shared in common between unexposed (BS90) and *S. mansoni* sporocyst-encapsulated (BS90-SP) hemocyte groups, while 35 and 32 unique proteins were identified in BS90 and BS90-SP hemocyte samples, respectively (Figure 3B). Similar to the NMRI treatment groups, 44 of the identified common proteins met the 4-fold minimum threshold difference (Table 3). However, in contrast to susceptible snail hemocytes, approximatively the same number of sporocyst-encapsulating BS-90 cell proteins were observed to be up and down-regulated in categories such as protein processing, metabolism, signaling, redox or immune-related proteins, with 59% of the proteins exhibiting a 4-fold decrease in presence of parasite.

**Table 3:** Proteins shared in common between control BS90 and sporocyst-encapsulating (BS90-SP) hemocytes that present at least a 4-fold difference in total normalized spectral counts.

**Common BS90 vs. BS90-SP: 4 fold difference**				
**NCBI acc. no**.	**Protein identification**	**BS90**	**BS90-SP**	**Function**
**IMMUNE-RELATED**				
XP_013095968.1	*/Proteasome subunit alpha type-5-like [*Aplysia californica*] XP_005110071.1 (91%)	3.938	0.94133	Proteasome
XP_013066054.1	Sequestosome-1-like [Bg]	7.4474	1.0082	Autophagy
XP_013066053.1	*/Sequestosome-1-like [*Limulus polyphemus*] XP_013792674.1(36%)	29.823	4.0329	Autophagy
XP_013064208.1	Reelin-like [Bg]	20.299	2.8909	Lipoprotein receptor binding
XP_013079231.1	N-acetylgalactosamine kinase-like [Bg]	0.9309	3.8991	Galactose binding
XP_013062216.1	26S proteasome non-ATPase regulatory subunit 12-like [Bg]	1.0381	4.7735	Proteasome
**REDOX**				
XP_013075526.1	Glutathione S-transferase 7 [Bg]	8.6998	2.0164	Detoxification of ROS
XP_013089814.1	Alcohol dehydrogenase class-3-like [Bg]	3.938	0.94133	Redox
XP_013077425.1	Ferric-chelate reductase 1 [Bg]	4.8689	1.0082	Redox
XP_013073942.1	Aldehyde dehydrogenase family 16 member A1-like [Bg]	10.883	1.94953	Redox
XP_013086335.1	*/Neurexin-1 [Aplysia californica] XP_012941838.1 (48%)	20.621	4.8404	Redox
XP_013069507.1	*/Phytanoyl-CoA dioxygenase 2-like [*Saccoglossus kowalevskii*] XP_006814546.1 (56%)	1.8619	7.7982	Fatty acid alpha-oxidation
XP_013084558.1	Dual oxidase-like [Bg]	0.9309	4.0329	Generates ROS
**APOPTOSIS AND REGULATION**				
XP_013091656.1	Apoptosis-inducing factor 3-like isoform X1 [Bg]	7.6617	1.0082	Pro-apoptosis
XP_013062216.1	26S proteasome non-ATPase regulatory subunit 12-like [Bg]	1.0381	4.7735	Proteasome
XP_013084356.1	Acidic leucine-rich nuclear phosphoprotein 32 family member B-like [Bg]	1.0381	8.8064	Anti-apoptotic
**STRESS RESPONSE**				
XP_013094041.1	Universal stress protein Slr1101-like [Bg]	3.8309	0.94133	Response to stress
**SIGNALING**				
XP_013069708.1	Protein Mo25-like [Bg]	0.9309	3.7653	Calcium binding protein
**CELL ADHESION/MOVEMENT**				
XP_013073410.1	Sushi, von Willebrand factor A, EGF /pentraxin domain-containing protein 1-like[Bg]	4.8689	0.94133	Cell attachment process
XP_013092731.1	Triple functional domain protein-like, partial [Bg]	4.1522	1.0082	Actin remodeling
XP_013068207.1	Myosin heavy chain, striated muscle-like isoform X1 [Bg]	5.1903	1.0082	Muscle contraction
**TRANSCRIPTION/TRANSLATION PROCESS**				
XP_013089238.1	60S Ribosomal protein L36-like [Bg]	3.938	0.94133	Pibosomal
XP_013082582.1	Eukaryotic translation initiation factor 3 subunit K-like [Bg]	4.9761	0.94133	Translation regulation
XP_013096529.1	Eukaryotic peptide chain release factor subunit 1 [Bg]	3.8309	0.94133	Translation regulation
XP_013089019.1	Protein disulfide-isomerase A4-like [Bg]	4.0451	1.0082	Protein folding
XP_013093243.1	*/DNA ligase 1-like isoform X3 [Bg] XP_013093256.1 (100%)	16.683	3.8322	DNA binding/ repair
XP_013078837.1	40S Ribosomal protein S12-like [Bg]	1.0381	5.7817	Ribosomal
XP_013061577.1	40S Ribosomal protein S25-like [Bg]	1.0381	4.8404	Ribosomal
XP_013073661.1	Cleavage and polyadenylation specificity factor subunit 5-like [Bg]	1.969	9.6809	Polyadenylation
XP_013080627.1	Bifunctional glutamate/proline–tRNA ligase-like [Bg]	1.0381	4.8404	Translation regulation
XP_013060775.1	Calumenin-like [Bg]	0.9309	6.5893	Calcium binding
**METABOLISM**				
XP_013093293.1	Trifunctional nucleotide phosphoesterase protein YfkN-like, partial [Bg]	4.7618	0.94133	Nucleotide catabolic process
XP_013083683.1	Sulfotransferase family cytosolic 1B member 1-like, partial [Bg]	4.9761	1.0082	Lipid metabolism
XP_013095124.1	Stomatin-like protein 2, mitochondrial [Bg]	4.9761	1.0082	Lipid binding
XP_013069854.1	Phosphonoacetaldehyde hydrolase-like [Bg]	0.9309	8.8064	Phosphonate catabolism
XP_013070092.1	Ethanolamine-phosphate cytidylyltransferase-like isoform X1 [Bg]	0.9309	3.8991	Lipid metabolism
XP_013094998.1	Enoyl-CoA hydratase, mitochondrial-like isoform X1 [Bg]	1.0381	4.7735	Lipid metabolism
XP_013075978.1	Perilipin-2-like [Bg]	1.0381	4.8404	Lipid metabolism
**PROTEIN SORTING AND TRAFFICKING**				
XP_013077091.1	ATP synthase subunit g, mitochondrial-like, partial [Bg]	4.9761	0.94133	Proton transporter
XP_013085659.1	Coatomer subunit beta'-like [Bg]	5.7998	1.0082	Protein transport
XP_013086081.1	*/Tripartite motif-containing protein 3-like isoform X1 [*Pomacea canaliculata*] (25%)	4.0451	0.94133	Vesicular trafficking
XP_013073443.1	dynamin-1-like [Bg]	8.0902	1.0082	Endocytosis.
**MISC**				
XP_013062313.1	Unch. prot. LOC106051655 [Bg]	4.0451	28.369	Unknown
XP_013089315.1	*/Caffeoyl-CoA O-methyltransferase 1 [Bg] XP_013087214.1 (59%)	0.9309	3.96603	Methyl transferase

NCBI accession numbers and protein identification are listed. Color backgrounds indicate an increased spectral count for proteins in the corresponding treatment. Uncharacterized proteins (*/) are followed by their closest NCBI Blastp IDs and ID percentages.

Contrary to the near complete shutdown of protein processing/metabolism in larval-encapsulating NMRI hemocytes, we observed an increase in 40S ribosomal proteins and others involved in mRNA/tRNA processing in BS-90 capsules indicating a marked enhancement of cellular translation processes in response to contact with encapsulated larvae (Table 3). By contrast, members of the argonaute family proteins (piwi and argonaute2), potentially involved in gene expression regulation via mi/piRNA were decreased in presence of sporocysts, suggesting a decrease in transcription mechanisms. Apoptosis also appears to be downregulated with a decrease in apoptosis-inducing factor 3, and a concurrent increase in an anti-apoptotic protein (acidic leucine-rich nuclear phosphoprotein 32), suggesting an overall reduction of the apoptosis mechanism in presence of parasites. Host modulation of hemocyte apoptosis in response to direct contact with the parasite, would likely prevent premature cell death in effector hemocytes thereby sustaining a more effective immune response. Interestingly, like NMRI hemocytes, sequestosome 1/p62 also was sharply down-regulated in sporocyst-encapsulated BS-90 cells (Table 3) suggesting that, although clearly a response to larval contact, hemocytes apparently do not require this protein for their cytotoxic activity. Upregulation of other immune-related genes such as T-complex protein 1, BPI (Table 1) and N-acetylgalactosamine kinase, involved in GalNAc binding interactions (Table 3) would be consistent with a functioning immune system.

The snail's redox system plays a central role, not only in maintaining the host's own redox balance, but as a primary effector response against larval helminths through production of ROS and reactive nitrogen species (RNS) (53). In response to sporocysts, encapsulating BS-90 hemocytes exhibited both up- and down-regulation of redox proteins. Those showing significant decreases include glutathione S-transferase (GST) 7, ferric-chelate reductase 1, aldehyde dehydrogenase family (Table 3), as well as glutarate-semialdehyde, isocitrate dehydrogenase and microsomal GST 3 found expressed as unique proteins (Table 1). However, down-regulation of these redox-related proteins is countered by increases in hydrogen peroxide-generating dual oxidase and phytanoyl-CoA dioxygenase 2 (Table 3), and uniquely expressed Cu-Zn superoxide dismutase (SOD1), D-arabinono-1,4-lactone oxidase and laccase-3 in the presence of parasites (Table 1). Dual oxidase, a transmembrane protein and member of the NADPH oxidase family, produces ROS, specifically hydrogen peroxide (54). Proteins similarly involved in H_2_O_2_ production include the peroxisomal enzyme, phytanoyl-CoA dioxygenase, SOD 1, and D-arabinono-1,4-lactone oxidase. Laccase3 is a phenoloxidase-related enzyme known to be active in *B. glabrata* hemolymph (55) and involved in molluscan immune defenses (56). The involvement of ROS in mediating killing of *S. mansoni* sporocysts is well-documented (53), as is the association of SOD1 gene expression with snail resistance to larval schistosome infection (57). Our finding that ROS-associated enzymes are upregulated in resistant snail hemocytes during encapsulation reactions is consistent with these reported observations.

Overall, the proteomic response of BS-90 snail hemocytes to the presence of sporocysts appears to be more balanced than in the susceptible snails in terms of maintaining metabolic functions, while at the same time mounting a potentially robust immune response against encapsulated larvae through upregulated expression of both shared and unique proteins associated with cellular metabolism and various immune functions.

### Host strain comparison of NMRI and BS-90 *B. glabrata* hemocyte responses to sporocysts

Paired comparison of the proteomic responses of *B. glabrata* strains to the presence of *S. mansoni* sporocysts (NMRI-SP vs. BS-90-SP) revealed 76 unique proteins in the BS90-SP group, 29 unique proteins in NMRI-SP group and 642 commonly expressed proteins (Figure 3C). A total of 39 proteins shared between the two strains, presented a minimum of 4-fold differences. A large majority of these proteins (9/39, 77%) were found to be decreased/downregulated in NMRI-SP compared to BS90-SP suggesting either a suppressive parasite effect on the susceptible snail host and/or an enhanced response by the resistant host strain to encapsulated parasite (Table 4). As for unique proteins of interest, all of those found earlier in BS90-SP capsules when compared to BS-90 controls were also uniquely present in BS90-SP when compared to NMRI-SP, suggesting either a lack/delay in the recognition of, or response to, sporocysts by NMRI hemocytes at this time point and/or a parasite-mediated suppression exclusively in hemocyte of the NMRI snail strain. In total, we observed 30 proteins uniquely expressed in BS90-SP compared to NMRI-SP that appear to be constitutively present in hemocytes of the BS-90 *B. glabrata* strain, which could be an important contributing factor to earlier parasite recognition/response in resistant snails.

**Table 4:** Proteins shared in common between sporocyst (SP)-encapsulating hemocytes of susceptible NMRI and resistant BS90 *B. glabrata* that present at least a 4-fold difference in total normalized spectral counts.

**BS90-SP vs. NMRI-SP: 4 fold difference**				
**NCBI acc. no**.	**Protein identification**	**BS90-SP**	**NMRI-SP**	**Function**
**IMMUNE RELATED**				
XP_013070436.1	Syntenin-1-like isoform X1 [Bg]	3.96603	0.95609	Trafficking/ immunomodulation
XP_013067749.1	Lysosomal alpha-mannosidase-like, partial [Bg]	3.8991	0.95609	Oligosaccharide catabolism
XP_013070669.1	Lipopolysaccharide-binding protein-like [Bg]	14.923	1.0127	Innate immune response
XP_013078814.1	*/ Proteoglycan 4-like [Aplysia californica] XP_005092461.1 (45%)	8.6727	1.96879	Immune response
XP_013079231.1	N-acetylgalactosamine kinase-like [Bg]	3.8991	0.95609	Galactose binding
XP_013064208.1	Reelin-like [Bg]	2.8909	16.3097	Lipoprotein receptor binding
**REDOX**				
XP_013077724.1	*/Carbonyl reductase [NADPH] 1-like [Bg] XP_013077725.1 (74%)	19.6296	0.95609	Redox
XP_013080583.1	Thioredoxin reductase 1, cytoplasmic-like isoform X1 [Bg]	7.99883	0.95609	Redox
XP_013069507.1	*/Phytanoyl-CoA dioxygenase domain-containing protein 1-like [*Pomacea canaliculata*] XP_025105244.1 (70%)	7.7982	1.0127	Redox
XP_013060614.1	*/ Hemoglobin type 1 [Bg] CAJ44466.1 (58%)	13.179	1.9122	O_2_ binding
XP_013062383.1	*/Hemoglobin type 1 [Bg] CAJ44466.1 (82%)	44.5771	8.718	O_2_ binding
XP_013062584.1	*/ Hemoglobin type 2 CAJ44467.1 (99%)	103.608	13.5552	O_2_ binding
XP_013075721.1	D-amino-acid oxidase-like [Bg]	1.8827	8.8879	Redox
XP_013062841.1	Prolyl 4-hydroxylase subunit alpha-1-like isoform X2 [Bg]	0.94133	5.7931	Redox
XP_013080374.1	Electron transfer flavoprotein subunit alpha, mitochondrial-like [Bg]	2.0164	8.8879	Redox
**APOPTOSIS**				
XP_013066053.1	*/sequestosome-1-like [*Limulus polyphemus*] XP_013792674.1 (36%)	4.0329	0.95609	Immune/Autophagy
XP_013091656.1	Apoptosis-inducing factor 3-like isoform X1 [Bg]	1.0082	4.8937	Apoptosis
**STRESS RESPONSE**				
XP_013075913.1	Endoplasmin-like [Bg]	3.8322	0.95609	Chaperone
XP_013079911.1	Heterochromatin protein 1-binding protein 3-like isoform X1 [Bg]	4.8404	0.95609	Cellular response to hypoxia
XP_013065461.1	Protein deglycase DJ-1zDJ-1-like [Bg]	7.932	1.96879	Chaperone/Stress response
**SIGNALING**				
XP_013077041.1	Receptor-type tyrosine-protein phosphatase alpha-like, partial [Bg]	4.7066	0.95609	MAPK cascade
XP_013077039.1	*/ Receptor-type tyrosine-protein phosphatase alpha-like [Bg] XP_013063115.1 (42%)	5.7817	0.95609	MAPK cascade
XP_013081619.1	Serine/threonine-protein kinase PAK 2-like [Bg]	13.6468	1.96879	Signaling
XP_013087690.1	Formin-like protein CG32138 [Bg]	6.79	0.95609	Rac GTPase binding
XP_013088191.1	Guanine nucleotide-binding protein G(o) subunit alpha [Bg]	4.7735	0.95609	GTP binding
**TRANSCRIPTION/TRANSLATION PROCESS**				
XP_013061577.1	40S Ribosomal protein S25-like [Bg]	4.8404	0.95609	Translation initiation
XP_013070062.1	Protein LZIC-like [Bg]	4.9074	1.0127	Transcription factor
XP_013062948.1	RNA-binding protein lark-like [Bg]	5.7148	0.95609	Translation regulation
XP_013063676.1	*/ Nucleolin-like [*Aplysia californica*] XP_005109234.1 (31%)	5.0411	0.95609	Translation regulation
XP_013063201.1	Histone H1-delta-like [Bg]	5.8487	1.0127	Transcription regulation
XP_013084934.1	Heterogeneous nuclear ribonucleoprotein U-like protein 1 isoform X1 [Bg]	14.5881	1.96879	Transcription regulation
**METABOLISM/CATABOLISM**				
XP_013068834.1	3-Hydroxyacyl-CoA dehydrogenase type-2-like [Bg]	4.7735	0.95609	Lipid metabolism
XP_013072118.1	Glycogen debranching enzyme-like [Bg]	4.7735	0.95609	Catabolic glycogen process
XP_013062675.1	UTP–glucose-1-phosphate uridylyltransferase-like [Bg]	7.7313	0.95609	Glucose metabolism
XP_013081380.1	Fumarate hydratase class I, aerobic-like [Bg]	0.94133	3.881	Carbohydrate metabolism
XP_013069189.1	2-Aminoethylphosphonate–pyruvate transaminase-like [Bg]	1.94953	8.8313	Phosphonate Catabolism
XP_013076947.1	Fatty acid synthase-like [Bg]	0.94133	4.837	Lipid metabolism
**MISC**				
XP_013088283.1	*/Cartilage matrix protein-like [*Aplysia californica*] XP_005102985.2 (50%)	5.98243	0.95609	Collagen binding
XP_013075555.1	ATP synthase subunit f, mitochondrial [Bg]	1.0082	6.9192	Proton transport

NCBI accession numbers and protein identification are listed. Color backgrounds indicate an increased spectral count for proteins in the corresponding treatment. Uncharacterized proteins (*/) are followed by their closest NCBI Blastp IDs and ID percentages.

Among those specific proteins with reduced expression in NMRI-SP compared to BS-90-SP, a high proportion were associated with immune, redox and cell signaling functions. For immune-related proteins, those exhibiting decreased expression include LPS binding protein (LBP), syntenin1, and lysosomal alpha-mannosidase (Table 4), as well as uniquely identified perilipin 2, BPI protein, and fibrinogen-related protein A precursor (FREP A precursor) (Table 1). As previously mentioned, LBP from snail plasma has been found to interact with the tegument and secretions of *S. mansoni* sporocysts (43) and therefore, differences may reflect lower NMRI plasma LBP concentrations and/or decreased hemocyte LBP synthesis. Syntenin 1 is an adapter protein, containing PDZ (postsynaptic density, discs large, zona-occludens) domains, allowing for multiprotein complex assembly (58) involved in various mechanisms including apoptosis regulation (eukaryotic translation initiation factor 5A) and immunity (alpha-2-macroglobulin) (59, 60). Alpha-mannosidase-like is a lysosomal hydrolase that, similar to other known hydrolases in *B. glabrata* hemolymph (61), can be released from hemocytes to degrade pathogen-associated glycans (62), while peripilin 2, a component of lipid droplets (LD), may potentially be involved in immune reactivity to microbial pathogens as shown in *Aedes* mosquitoes (63).

FREP A precursor protein, which presents a 96% identity with FREP 2.1, was found constitutively expressed solely in hemocytes of the BS-90 resistant strain. FREPs represent a diverse group of lectin PRRs (27, 64) that are closely linked to the snail's immune response against larval *S. mansoni* (65). Consistent with our current data, FREP2 transcripts were found to be dramatically increased following *S. mansoni* exposure of BS-90, but not susceptible M-line, snails (66). Although FREP2 is present in the plasma of both susceptible and resistant *B. glabrata* strains, and is reactive with glycoproteins [e.g., polymorphic mucins (67)] of larval *S. mansoni*, our finding of these immune proteins in BS90/BS-90-SP hemocyte capsules, but not those of NMRI/NMRI-SP, suggests a specific interaction of a FREP2 subfamily variant with BS-90 hemocytes during cell-cell or cell-parasite aggregation.

Similarly, we observed numerous redox proteins that were reduced or downregulated in NMRI-SP capsules when compared to those of BS90-SP, including SOD1, thioredoxin reductase 1, GST4, dual oxidase, laccase 3 and hemoglobins 1 and 2. H_2_O_2_ and NO production have previously been shown to be critical to *in vitro* killing of *S. mansoni* sporocysts in the snail (53). Hemocyte SOD1, especially important as it catalyzes the conversion of ${\text{O}}_{2}^{-}$ to H_2_O_2_ during encapsulation, has been shown to have differential activity and transcriptomic levels according to the resistance/susceptibility of selected snail strains (57). Similarly, dual oxidase and laccase 3, as described in the previous section, also are involved in H_2_O_2_ production and phenoxidase activity, respectively, thereby contributing to the innate immune defenses in the host (56). Thioredoxin1 (TRX1) and GST4 are antioxidants likely contributing to cellular redox homeostasis that would be required by the hemocytes to regulate their own production of ROS in response of the parasite, thereby preventing intracellular damage while also engaging in cytotoxic activities. In NMRI-SP encapsulations, a similar homeostatic mechanism may not be required since NMRI hemocytes were not found to release as much ROS in presence of parasites. GST was previously reported to be upregulated in snails exposed to sublethal doses of molluscicides, suggesting a stress-related response more than a specific immune response to a pathogen (68).

One explanation for the comparatively weak response of encapsulating NMRI hemocytes to sporocysts is that susceptible snail strains are, in general, immunologically inferior to the resistant strains. However, previous studies have shown that a single strain of *B. glabrata* can be both susceptible to one strain of *S. mansoni* while at the same time resistant to a different *S. mansoni* strain (34, 35), indicating that parasite infectivity factors are equally important in determining host-parasite compatibility as the genetic makeup of the snail host (2–4). This also holds true for snails confronted with other non-schistosome pathogens. For example, *Echinostoma paraensei* has been shown to infect both R and S strains of *B. glabrata* (66, 69), while bacterial pathogens *Paenibacillus* (70) and *Vibrio parahaemolyticus* (71) infect all exposed *B. glabrata* strains regardless of their schistosome-susceptibility status. This strongly suggests that the S and R phenotypes exhibited by these model *B. glabrata/S. mansoni* systems are host/parasite strain-specific, and generalizations as to their immune status or susceptibility to other pathogens cannot be made.

Finally, we demonstrated that NMRI and BS-90 snail hemocytes, irrespective of the presence or absence of sporocysts, consistently differed in their levels of hemoglobins (Hb)1 and 2, with BS-90 capsules containing more protein (based on fourfold higher total normalized spectral counts) than NMRI (Supplementary Table 2). This difference is most likely due to different levels of plasma Hb binding to hemocytes (72) and/or sporocysts (73, 74), and not due to the synthesis and differential expression by encapsulating cells. The immune-relevance of hemocyte-bound Hb is presently unclear. However, it has previously been demonstrated that snail Hb was toxic to schistosome sporocysts *in vitro* (75) and that reactions of Hb-conjugated Fe^2+^ with NO can lead to the generation of peroxynitrites, a powerful RNS with potential pathogen killing capacity (76). Recently, a role for Hb2 as a parasite immune “sensing” protein has been postulated (74), thereby opening up the possibility that higher constitutive levels of cell-bound Hb (as shown in the present study) may contribute to an early response/killing by resistant BS-90 snail hemocytes in this model system. In addition to Hb, other *B. glabrata* plasma proteins with sporocyst membrane-binding activity (43, 67) were found to be associated with capsular hemocytes including several with known or proposed immune function: LBP, fibrinogen-like protein A (FREP 2.1), FREP F precursor, alpha-amylase and an actin2 protein (4, 50, 64, 67, 74). Although hemocytes themselves may be the source of these proteins, it is more likely that they are of plasma origin and possess hemocyte and/or sporocyst binding activity.

In summary, we have investigated the proteomic response of *B. glabrata* hemocytes during *in vitro* encapsulation of *S. mansoni* sporocysts by comparing the proteomic profiles of reactive hemocytes both within and between susceptible and resistant snail strains. In the susceptible NMRI *B. glabrata* strain, we observed a dramatic downregulation of proteins during parasite encapsulation, many of which are related to immune, redox and autophagic function. Of the few upregulated proteins, one (arginase 1) appeared to be induced by the parasite to favor its survival during encapsulation. A more balance proteomic response was exhibited by resistance BS-90 snails, supporting a more robust immune capacity. These included several mechanisms involved in parasite killing such as an upregulation of proteins associated with H_2_O_2_ production (SOD1, dual oxidase) and antioxidants to counter intracellular oxidative damage to hemocytes. Interestingly, the NMRI/BS-90 strain comparison highlighted the potential importance of hemocyte proteins constitutively expressed in the resistant snails, but absent in the susceptible strain. These included FREP2, mannosidase and a potential NO-dependent role of hemoglobin in the resistant snail. The constitutive presence of these, and other larval-responsive, immune-related proteins provide important insights into effector mechanisms of encapsulating hemocytes that characterize the resistance phenotype in this model host-parasite system.

## Author contributions

MC and TY developed study concept and, with ND, created the experimental design. MC performed experiments. MC, ND, X-JW, and UB-W data analysis and interpretation. GS generated proteomic data. GS, ND, and UB-W bioinformatic analyses. ND, MC, GS, and TY manuscript writing and editing. ND and TY critical review and final editing. All authors approved the final submitted draft.

### Conflict of interest statement

The authors declare that the research was conducted in the absence of any commercial or financial relationships that could be construed as a potential conflict of interest. The reviewer CA declared a past co-authorship with one of the authors TY, to the handling Editor.

## Supplementary material

Supplementary Table 1 : *Biomphalaria glabrata* hemocyte proteins identified in two replicate experiments. Each entry lists the identified protein, NCBI accession number, molecular weight and normalized spectral counts for each treatment: control hemocyte capsules (BS90, NMRI), hemocyte capsules with sporocysts (BS90-SP, NMRI-SP). Numbers 1 and 2 (e.g., BS90 1) represent the first and second independent replicate, respectively.

Supplementary Table 2 : List of proteins showing high identities to *Biomphalaria glabrata* hemoglobins (Hb) 1 or 2. NCBI accession numbers and molecular weights are listed for each entry (NMRI, NMRI-SP, BS90, BS90-SP) with their corresponding total normalized spectral counts. Initial protein identifications were returned as “uncharacterized protein.” However, subsequent BlastP searches of these sequences revealed highest identities to *B. glabrata* Hb 1 and 2.

## References

[1] Adema CM Loker ES. Digenean-gastropod host associations inform on aspects of specific immunity in snails. Dev Comp. Immunol. (2015) 48:275–283. 10.1016/j.dci.2014.06.014 25034871 PMC4258543

[2] Coustau C Gourbal B Duval D Yoshino TP Adema CM Mitta G. Advances in gastropod immunity from the study of the interaction between the snail *Biomphalaria glabrata* and its parasites: a review of research progress over the last decade. Fish Shellfish Immunol. (2015) 46:5–16. 10.1016/j.fsi.2015.01.036 25662712

[3] Mitta G Gourbal B Grunau C Knight M Bridger JM Théron A. The compatibility between *Biomphalaria glabrata* snails and *Schistosoma mansoni*: An increasingly complex puzzle. Adv Parasitol. (2017) 97:112–44. 10.1016/bs.apar.2016.08.006 28325369

[4] Pila EA Li H Hambrook JR Wu X Hanington PC. Schistosomiasis from a snail's perspective: advances in snail immunity. Trends Parasitol. (2017) 33:845–57. 10.1016/j.pt.2017.07.006 28803793

[5] Yoshino TP Gourbal B Théron A *Schistosoma* sporocysts. In: Jamieson BGM, editor. Schistosoma: Biology, Pathology and Control. Boca Raton, FL: CRC Press (2017). p. 118–48.

[6] Kassim OO Richards CS. Host reactions in *Biomphalaria glabrata* to *Schistosoma mansoni* miracidia, involving variations in parasite strains, numbers and sequence of exposures. Int J Parasitol. (1979) 9:565–70. 541169 10.1016/0020-7519(79)90013-4

[7] Loker ES Bayne CJ. *In vitro* encounters between *Schistosoma mansoni* primary sporocysts and hemolymph components of susceptible and resistant strains of *Biomphalaria glabrata*. Am J Trop Med Hyg. (1982) 31:999–1005. 7125067 10.4269/ajtmh.1982.31.999

[8] Bayne CJ Buckley PM DeWan PC Macrophage-like hemocytes of resistant *Biomphalaria glabrata* are cytotoxic for sporocysts of *Schistosoma mansoni in vitro*. J Parasitol. (1980) 66:413–19. 7391885

[9] Basch PF DiConza JJ. The miracidium-sporocyst transition in *Schistosoma mansoni*: surface changes *in vitro* with ultrastructural correlation. J Parasitol. (1974) 60:935–41. 10.2307/3278518 4436765

[10] Bayne CJ Buckley PM DeWan PC. *Schistosoma mansoni*: cytotoxicity of hemocytes from susceptible snail hosts for sporocysts in plasma from resistant *Biomphalaria glabrata*. Exp Parasitol. (1980) 50:409–16. 7428914 10.1016/0014-4894(80)90043-0

[11] Allan ERO Gourbal B Dores CB Portet A Bayne CJ Blouin MS. Clearance of schistosome parasites by resistant genotypes at a single genomic region in *Biomphalaria glabrata* snails involves cellular components of the hemolymph. Internatl J Parasitol. (2018) 48:387–93. 10.1016/j.ijpara.2017.08.008 29137971 PMC5893386

[12] Tennessen JA Théron A Marine M Yeh JY Rognon A Blouin MS. Hyperdiverse gene cluster in snail host conveys resistance to human schistosome parasites. PLoS Genet (2015) 11:e1005067. 10.1371/journal.pgen.1005067 25775214 PMC4361660

[13] Allan ERO Tennessen JA Bollmann SR Hanington PC Bayne CJ Blouin MS. Schistosome infectivity in the snail, *Biomphalaria glabrata*, is partially dependent on the expression of Grctm6, a Guadeloupe Resistance Complex protein. PLoS Negl Trop Dis (2017) 11:e0005362. 10.1371/journal.pntd.0005362 28158185 PMC5310918

[14] Mitta G Galinier R Tisseyre P Allienne J-F Girerd-Chambaz Y Guillou F. Gene discovery and expression analysis of immune-relevant genes from *Biomphalaria glabrata* hemocytes. Dev Comp Immunol. (2005) 29:393–407. 10.1016/j.dci.2004.10.002 15707661

[15] Lockyer AE Spinks J Noble LR Rollinson D Jones CS. Identification of genes involved in interactions between *Biomphalaria glabrata* and *Schistosoma mansoni* by suppression subtractive hybridization. Mol Biochem Parasitol. (2007) 151:18–27. 10.1016/j.molbiopara.2006.09.009 17081633 PMC1852639

[16] Lockyer AE Spinks J Kane RA Hoffman KF Fitzpatrick JM Rollinson D. *Biomphalaria glabrata* transcriptome: cDNA microarray profiling identifies resistant- and susceptible-specific gene expression in haemocytes from snail strains exposed to *Schistosoma mansoni*. BMC Genomics (2008) 9:634. 10.1186/1471-2164-9-634 19114004 PMC2631019

[17] Kenny NJ Truchado-García M Grande C. Deep, multi-stage transcriptome of the schistosomiasis vector *Biomphalaria glabrata* provides platform for understanding molluscan disease-related pathways. BMC Infect Dis. (2016) 16:618. 10.1186/s12879-016-1944-x 27793108 PMC5084317

[18] Raghavan N Miller AN Gardner M FitzGerald PC Kerlavage AR Johnston DA. Comparative gene analysis of *Biomphalaria glabrata* hemocytes pre- and post-exposure to miracidia of *Schistosoma mansoni*. Mol Biochem Parasitol. (2003) 126:181–91. 10.1016/S0166-6851(02)00272-4 12615317

[19] Guillou Mitta Galinier Coustau C. Identification and expression of gene transcripts generated during an anti-parasitic response in *Biomphalaria glabrata*. Dev Comp Immunol. (2007) 31:657–71. 10.1016/j.dci.2006.10.001 17166585

[20] Adema CM Hanington PC Lun CM Rosenberg GH Aragon AD Stout BA. Differential transcriptomic responses of *Biomphalaria glabrata* (Gastropoda, Mollusca) to bacteria and metazoan parasites, *Schistosoma mansoni* and *Echinostoma paraensei* (Digenea, Platyhelminthes). Mol Immunol. (2010) 47:849–60. 10.1016/j.molimm.2009.10.019 19962194 PMC2814977

[21] Lockyer AE Emery AM Kane RA Walker AJ Mayer CD Mitta G. Early differential gene expression in haemocytes from resistant and susceptible *Biomphalaria glabrata* strains in response to *Schistosoma mansoni*. PLoS ONE (2012) 7:e51102. 10.1371/journal.pone.0051102 23300533 PMC3530592

[22] Zahoor Z Lockyer AE Davies AJ Kirk RS Emery AM Rollinson D. Differences in the gene expression profiles of haemocytes from schistosome-susceptible and -resistant *Biomphalaria glabrata* exposed to *Schistosoma mansoni* excretory-secretory products. PloS ONE (2014) 9:e93215. 10.1371/journal.pone.0093215 24663063 PMC3963999

[23] Bouchut A Sautiere PE Coustau C Mitta G. Compatibility in the *Biomphalaria glabrata/Echinostoma caproni* model: Potential involvement of proteins from hemocytes revealed by a proteomic approach. Acta Trop (2006) 98:234–46. 10.1016/j.actatropica.2006.05.007 16792992

[24] Bender RC Goodall CP Blouin MS Bayne CJ. Variation in expression of *Biomphalaria glabrata* SOD1: a potential controlling factor in susceptibility/resistance to *Schistosoma mansoni*. Dev Comp Immunol. (2007) 31:874e878. 10.1016/j.dci.2006.12.005 17292470

[25] Zahoor Z Davies AJ Kirk RS Rollinson D Walker AJ Larval excretory-secretory products from the parasite *Schistosoma mansoni* modulate HSP 70 protein expression in defence cells of its snail host, *Biomphalaria glabrata*. Cell Stress Chaperones (2010) 15:639–50. 10.1007/s12192-010-0176-z 20182834 PMC3006636

[26] Ittiprasert W Knight M. Reversing the resistance phenotype of the *Biomphalaria glabrata* snail host *Schistosoma mansoni* infection by temperature modulation. PLoS Pathog. (2012) 8:e1002677. 10.1371/journal.ppat.1002677 22577362 PMC3343117

[27] Hanington PC Forys MA Dragoo JW Zhang SM Adema CM Loker ES. Role for a somatically diversified lectin in resistance of an invertebrate to parasite infection. Proc Natl Acad Sci USA. (2010) 107:21087–92. 10.1073/pnas.1011242107 21084634 PMC3000291

[28] Hanington PC Forys MA Loker ES. A somatically diversified defense factor, FREP3, is a determinant of snail resistance to schistosome infection. PLoS Negl Trop Dis. (2012) 6:e1591. 10.1371/journal.pntd.0001591 22479663 PMC3313920

[29] Portet A Galinier R Pinaud S Portela J Nowacki F Gourbal B. BgTEP: An antiprotease involved in innate immune sensing in *Biomphalaria glabrata*. Front Immunol. (2018) 9:1206. 10.3389/fimmu.2018.01206 29899746 PMC5989330

[30] Baeza-Garcia A Pierce RJ Gourbal B Werkmeister E Colinet D Reichhart JM. Involvement of the cytokine MIF in the snail host immune response to the parasite *Schistosoma mansoni*. PLoS Pathog. (2010) 6:e1001115. 10.1371/journal.ppat.1001115 20886098 PMC2944803

[31] Galinier R Portela J Moné Y Allienne JF Henri H Delbecq S. Biomphalysin, a new pore-forming toxin involved in *Biomphalaria glabrata* immune defense against *Schistosoma mansoni*. PLoS Pathog. (2013) 9:e1003216. 10.1371/journal.ppat.1003216 23555242 PMC3605176

[32] Pila EA Tarrabain M Kabore AL Hanington PC. A Novel Toll-Like receptor (TLR) influences compatibility between the gastropod *Biomphalaria glabrata*, and the digenean trematode *Schistosoma mansoni*. PLoS Pathog. (2016) 12:e1005513. 10.1371/journal.ppat.1005513 27015424 PMC4807771

[33] Pila EA Gordy MA Phillips VK Kabore AL Rudko SP Hanington PC. Endogenous growth factor stimulation of hemocyte proliferation induces resistance to *Schistosoma mansoni* challenge in the snail host. Proc Natl Acad Sci USA. (2016) 113:5305–10. 10.1073/pnas.1521239113 27114544 PMC4868488

[34] Théron A Rognon A Gourbal B Mitta G. Multi-parasite host susceptibility and multi-host parasite infectivity: a new approach of the *Biomphalaria glabrata/Schistosoma mansoni* compatibility polymorphism. Infect Genet Evol. (2014) 26:80–8. 10.1016/j.meegid.2014.04.025 24837670

[35] Galinier R Roger E Moné Y Duval D Portet A Pinaud S. A multistrain approach to studying the mechanisms underlying compatibility in the interaction between *Biomphalaria glabrata* and *Schistosoma mansoni*. PLoS Negl Trop Dis. (2017) 11:e0005398. 10.1371/journal.pntd.0005398 28253264 PMC5349689

[36] Yoshino TP Laursen JR. Production of *Schistosoma mansoni* daughter sporocysts from mother sporocysts maintained in synxenic culture with *Biomphalaria glabrata* embryonic Bge cells. J Parasitol. (1995) 81:714–22. 10.2307/3283960 7472861

[37] Dinguirard N Heinemann C Yoshino TP Mass isolation and *in vitro* cultivation of intramolluscan stages of the human blood fluke *Schistosoma mansoni*. J Vis Exp. (2018) 131:e56345 10.3791/56345 PMC5908647 29364215

[38] Chernin E. Observations on hearts explanted *in vitro* from the snail *Australorbis glabratus*. J Parasitol. (1963) 49:353–64. 10.2307/3275797 14020610

[39] Sminia T Barendsen LA Comparative morphological and enzyme biochemical study of blood cells of the freshwater snails *Lymnaea stagnalis, Biomphalaria glabrata*, and *Bulinus truncates*. J Morphol. (1980) 165:31–9. 10.1002/jmor.1051650104 30153712

[40] Hahn UK Bender RC Bayne CJ. Killing of *Schistosoma mansoni* sporocysts by hemocytes from resistant *Biomphalaria glabrata*: role of reactive oxygen species. J Parasitol. (2001) 87:292–9. 10.1645/0022-3395(2001)087[0292:KOSMSB]2.0.CO;2 11318558

[41] Nesvizhskii AI Keller A Kolker E Aebersold R. A statistical model for identifying proteins by tandem mass spectrometry. Anal Chem. (2003) 75:4646–58. 10.1021/ac0341261 14632076

[42] Sievers F Wilm A Dineen D Gibson TJ Karplus K Li W. Fast, scalable generation of high-quality protein multiple sequence alignments using Clustal Omega. Mol Syst Biol. (2011) 7:539. 10.1038/msb.2011.75 21988835 PMC3261699

[43] Wu XJ Dinguirard N Sabat G Lui HD Gonzalez L Gehring M. Proteomic analysis of *Biomphalaria glabrata* plasma proteins with binding affinity to those expressed by early developing larval *Schistosoma mansoni*. PloS Pathog. (2017) 13:e1006081. 10.1371/journal.ppat.1006081 28520808 PMC5433772

[44] Staczek S Zdybicka-Barabas A Mak P Sowa-Jasiłek A Kedracka-Krok S Jankowska U. Studies on localization and protein ligands of *Galleria mellonella* apolipophorin III during immune response against different pathogens. J Insect Physiol. (2018) 105:18–27. 10.1016/j.jinsphys.2017.12.009 29289504

[45] Zhang SM Loker ES Sullivan JT. Pathogen-associated molecular patterns activate expression of genes involved in cell proliferation, immunity and detoxification in the amebocyte-producing organ of the snail *Biomphalaria glabrata*. Dev Comp Immunol. (2016) 56:25–36. 10.1016/j.dci.2015.11.008 26592964 PMC5335875

[46] Lippai M Low P. The role of the selective adaptor p62 and ubiquitin-like proteins in autophagy. Biomed Res Int. (2014) 2014:832704. 10.1155/2014/832704 25013806 PMC4075091

[47] Fujita K Srinivasula SM. TLR4-mediated autophagy in macrophages is a p62-dependent type of selective autophagy of aggresome-like induced structures (ALIS). Autophagy (2011) 7:552–4. 10.4161/auto.7.5.15101 21412052 PMC3127216

[48] Gan L Li L. Regulations and roles of the interleukin-1 receptor associated kinases (IRAKs) in innate and adaptive immunity. Immunol Res. (2006) 35:295. 10.1385/IR:35:3:295 17172653

[49] Queiroz FR Silva LM Jeremias WJ Babá ÉH Caldeira RL Coelho PMZ. Differential expression of small RNA pathway genes associated with the *Biomphalaria glabrata/Schistosoma mansoni* interaction. PLoS ONE (2017) 12:e0181483. 10.1371/journal.pone.0181483 28719649 PMC5515444

[50] Baron OL Deleury E Reichhart JM Coustau C. The LBP/BPI multigenic family in invertebrates: Evolutionary history and evidences of specialization in mollusks. Dev Comp Immunol. (2016) 57:20–30. 10.1016/j.dci.2015.11.006 26608112

[51] Guillou F Roger E Moné Y Rognon A Grunau C Théron A. Excretory-secretory proteome of larval *Schistosoma mansoni* and *Echinostoma caproni*, two parasites of *Biomphalaria glabrata*. Mol Biochem Parasitol. (2007) 155:45–56. 10.1016/j.molbiopara.2007.05.009 17606306

[52] Hahn UK Bender RC Bayne CJ Role of nitric oxide in killing of *Schistosoma mansoni* sporocysts by hemocytes from resistant *Biomphalaria glabrata*. J Parasitol. (2001) 87:778–85. 10.1645/0022-3395(2001)087[0778:IONOIK]2.0.CO;2 11534641

[53] Bayne CJ Hahn UK Bender RC. Mechanisms of molluscan host resistance and of parasite strategies for survival. Parasitology (2001) 123:S159–67. 10.1017/S0031182001008137 11769280

[54] Yang X Smith AA Williams MS Pal U. A dityrosine network mediated by dual oxidase and peroxidase influences the persistence of Lyme disease pathogens within the vector. J Biol Chem. (2014) 289:12813–22. 10.1074/jbc.M113.538272 24662290 PMC4007469

[55] Le Clec'h W Anderson TJC Chevalier FD. Characterization of hemolymph phenoloxidase activity in two *Biomphalaria* snail species and impact of *Schistosoma mansoni* infection. Parasit Vectors (2016) 9:32. 10.1186/s13071-016-1319-6 26797101 PMC4722754

[56] Luna-Acosta A Breitwieser M Renault T Thomas-Guyon H. Recent findings on phenoloxidases in bivalves. Mar Pollut Bull. (2017) 122:5–16. 10.1016/j.marpolbul.2017.06.031 28673617

[57] Goodall CP Bender RC Brooks JK Bayne CJ. *Biomphalaria glabrata* cytosolic copper/zinc superoxide dismutase (SOD1) gene: association of SOD1 alleles with resistance/susceptibility to *Schistosoma mansoni*. Mol Biochem Parasitol. (2006) 147:207–10. 10.1016/j.molbiopara.2006.02.009 16564582

[58] Fanning AS Anderson JM. PDZ domains: fundamental building blocks in the organization of protein complexes at the plasma membrane. J Clin Invest. (1999) 103:767–72. 10079096 10.1172/JCI6509 PMC408156

[59] Li AL Li HY Jin BF Ye QN Zhou T Yu XD. A novel eIF5A complex functions as a regulator of p53 and p53-dependent apoptosis. J Biol Chem. (2004) 279:49251–8. 10.1074/jbc.M407165200 15371445

[60] Tonganunt M Phongdara A Chotigeat W Fujise K. Identification and characterization of syntenin binding protein in the black tiger shrimp *Penaeus monodon*. J Biotechnol. (2005) 120:135–45. 10.1016/j.jbiotec.2005.06.006 16055222

[61] Granath WO Jr Yoshino TP. Lysosomal enzyme activities in susceptible and refractory strains of *Biomphalaria glabrata* during the course of infection with *Schistosoma mansoni*. J Parasitol. (1983) 69:1018–26. 6674450

[62] Mohandas A Cheng TC Cheng JB. Mechanism of lysosomal enzyme release from *Mercenaria* mercenaria granulocytes: a scanning electron microscope study. J Invertebr Pathol. (1985) 46:189–97. 4045216 10.1016/0022-2011(85)90148-x

[63] Barletta AB Alves LR Silva MC Sim S Dimopoulos G Liechocki S. Emerging role of lipid droplets in *Aedes aegypti* immune response against bacteria and Dengue virus. Sci Rep. (2016) 6:19928. 10.1038/srep19928 26887863 PMC4757862

[64] Zhang SM Adema CM Kepler TB Loker ES. Diversification of Ig superfamily genes in an invertebrate. Science (2004) 305:251–4. 10.1126/science.1088069 15247481

[65] Gordy MA Pila EA Hanington PC The role of FREPs fibrinogen-related proteins in the gastropod immune response. Fish Shellfish Immunol. (2015) 46:39–49. 10.1016/j.fsi.2015.03.005 25765166

[66] Hertel LA Adema CM Loker ES. Differential expression of FREP genes in two strains of *Biomphalaria glabrata* following exposure to the digenetic trematodes *Schistosoma mansoni* and *Echinostoma paraensei*. Dev Comp Immunol. (2005) 29:295–303. 10.1016/j.dci.2004.08.003 15859234

[67] Moné Y Gourbal B Duval D Du Pasquier L Kieffer-Jaquinod S Mitta G. A large repertoire of parasite epitopes matched by a large repertoire of host immune receptors in an invertebrate host/parasite model. PLoS Negl Trop Dis. (2010) 4:e813. 10.1371/journal.pntd.0000813 20838648 PMC2935394

[68] Zhang S-M Buddenborg SK Adema CM Sullivan JT Loker ES. Altered gene expression in the schistosome-transmitting snail *Biomphalaria glabrata* following exposure to niclosamide, the active ingredient in the widely used molluscicide Bayluscide. PLoS Negl Trop Dis. (2015) 9:e0004131. 10.1371/journal.pntd.0004131 26452273 PMC4599737

[69] Garcia JS Maldonado Junior A Bidau CJ Corrêa Ldos R Lanfredi RM Coelho PM. The effect of early infection with *Echinostoma paraensei* on the interaction of *Schistosoma mansoni* with *Biomphalaria glabrata* and *Biomphalaria tenagophila*. Mem Inst Oswaldo Cruz (2010) 105:499–503. 10.1590/S0074-02762010000400026 20721499

[70] Duval D Galinier R Mouahid G Toulza E Allienne JF Portela J A novel bacterial pathogen of *Biomphalaria glabrata*: a potential weapon for schistosomiasis control? PLoS Negl Trop Dis. (2015) 9:e0003489 10.1371/journal.pntd.0003489 25719489 PMC4342248

[71] Ducklow HW Tarraza HM Jr Mitchell R. Experimental pathogenicity of *Vibrio parahaemolyticus* for the schistosome-bearing snail *Biomphalaria glabrata*. Can J Microbiol. (1980) 26:503–6. 7378944 10.1139/m80-084

[72] Yoshino TP. Surface antigens of *Biomphalaria glabrata* (Gastropoda) hemocytes: occurrence of membrane-associated hemolymph-like factors antigenically related to snail hemoglobin. J Invertebr Pathol. (1983) 41:310–20. 6190950 10.1016/0022-2011(83)90248-3

[73] Bayne CJ Hull CJ. The host-parasite interface in molluscan schistosomiasis: biotin as a probe for sporocyst and hemocyte surface peptides. Vet Parasitol. (1988) 29:131–42. 10.1016/0304-4017(88)90121-5 3201703

[74] Tetreau G Pinaud S Portet A Galinier R Gourbal B Duval D. Specific pathogen recognition by multiple innate immune sensors in an invertebrate. Front Immunol. (2017) 8:1249. 10.3389/fimmu.2017.01249 29051762 PMC5633686

[75] Bender RC Bixler LM Lerner JP Bayne CJ. *Schistosoma mansoni* sporocysts in culture: host plasma hemoglobin contributes to *in vitro* oxidative stress. J Parasitol. (2002) 88:14–18. 10.1645/0022-3395(2002)088[0014:SMSICH]2.0.CO;2 12053954

[76] Coates CJ Decker H. Immunological properties of oxygen-transport proteins: hemoglobin, hemocyanin and hemerythrin. Cell Mol Life Sci. (2017) 74:293–317. 10.1007/s00018-016-2326-7 27518203 PMC5219038

